# Diagnostic Performance of Contrast-Enhanced Ultrasound (CEUS) in the Evaluation of Solid Renal Masses

**DOI:** 10.3390/medicina56110624

**Published:** 2020-11-19

**Authors:** Thomas Geyer, Vincent Schwarze, Constantin Marschner, Moritz L. Schnitzer, Matthias F. Froelich, Johannes Rübenthaler, Dirk-André Clevert

**Affiliations:** 1 Department of Radiology, Ludwig-Maximilians-University Munich, 81377 Munich, Germany; vincent.schwarze@med.uni-muenchen.de (V.S.); constantin.marschner@med.uni-muenchen.de (C.M.); moritz.schnitzer14@gmail.com (M.L.S.); johannes.ruebenthaler@med.uni-muenchen.de (J.R.); dirk.clevert@med.uni-muenchen.de (D.-A.C.); 2 Institute of Clinical Radiology and Nuclear Medicine, University Medical Center Mannheim, 68167 Mannheim, Germany; matthias.froelich@medma.uni-heidelberg.de

**Keywords:** CEUS, contrast-enhanced ultrasound, renal cell carcinoma, renal oncocytoma, renal angiomyolipoma, solid renal mass

## Abstract

*Background:* The present study aims to evaluate the diagnostic performance of contrast-enhanced ultrasound (CEUS) for discriminating between benign and malignant solid renal masses. *Methods:* 18 patients with histopathologically confirmed benign solid renal masses (11 oncocytomas, seven angiomyolipomas) as well as 96 patients with confirmed renal cell carcinoma (RCC) who underwent CEUS followed by radical or partial nephrectomy were included in this single-center study. CEUS examinations were performed by an experienced radiologist (EFSUMB Level 3) and included the application of a second-generation contrast agent. *Results:* Renal angiomyolipomas, oncocytomas, and renal cell carcinomas showed varying sonomorphological characteristics in CEUS. Angiomyolipomas showed heterogeneous echogenicity (57% hypo-, 43% hyperechoic), while all lesions showed rapid contrast-enhancement with two lesions also showing venous wash-out (29%). Notably, 9/11 oncocytomas could be detected in conventional ultrasound (64% hypo-, 9% hyper-, 9% isoechoic) and 2/11 only demarcated upon intravenous application of contrast agent (18%). All oncocytomas showed hyperenhancement in CEUS, venous wash-out was registered in 7/11 lesions (64%). *Conclusions:* In line with the current state of knowledge, no specific sonomorphological characteristics allowing for accurate distinction between benign and malignant solid renal masses in CEUS could be detected in our study.

## 1. Introduction

In recent decades, the number of unclear renal lesions incidentally detected on cross-sectional imaging or ultrasound has grown along with the more widespread use of medical imaging [1,2,3]. Conventional B-mode ultrasound is the most frequently used imaging modality for the initial classification of kidney lesions and allows for discriminating between cystic and solid renal lesions. Complicated renal cysts require dedicated contrast-enhanced computed tomography (CE-CT) or magnetic resonance imaging (MRI) for definitive characterization and the diagnosis of renal cell carcinoma (RCC) [4,5].

Recently, the use of contrast-enhanced ultrasound (CEUS) has established itself as a well-tolerated, highly accurate, and cost-effective imaging modality for diagnosing RCC [6,7,8]. CEUS represents a valuable alternative for characterizing indeterminate renal lesions especially in patients with decreased kidney function or allergic predispositions to gadolinium- or iodine-based contrast agents [5]. Unlike contrast-enhanced CT or MRI, ultrasound contrast agents remain confined to the intravascular system and can be used regardless of the renal or thyroid function [9,10].

In certain cases, however, differentiating benign solid renal masses from RCC remains a challenge. Renal oncocytoma, a benign parenchymal tumor with an incidence of 4–7% among all primary renal neoplasms [11], is known for its imaging characteristics similar to RCC in CE-CT [12], MRI [13], and CEUS [14,15,16]. Similar difficulties in the distinction between lipid-poor angiomyolipoma and renal cell carcinoma have been reported by several studies [17,18,19,20]. Since treatment recommendations and further patient management for renal oncocytoma or angiomyolipoma differ significantly from those for renal cell carcinoma [5], patients could benefit strongly from an adequate radiological differential diagnosis. 

Our study aimed to evaluate the diagnostic performance of CEUS of solid renal masses and potentially identify imaging features that might allow for distinguishing between benign and malignant solid renal lesions in the future.

## 2. Materials and Methods 

Our retrospective single-center study was approved by the local institutional ethical committee of the institutional review board (Ethics Committee, Medical Faculty, Ludwig Maximilian University of Munich; 17-087; date of approval: 14 March 2017). All contributing authors followed the ethical guidelines for publication in *Medicina*. All data used for this study were gathered according to the principles of the Declaration of Helsinki/Edinburgh in 2002. Before the CEUS examination, oral and written informed consent of all patients was obtained after potential risks and complications had been explained in detail. All examinations were performed by one experienced consultant radiologist (European Federation of Societies for Ultrasound in Medicine and Biology Level 3) using high-end ultrasound systems with CEUS-specific protocols (GE Healthcare LOGIQ E9, Chicago, IL, USA, Philips EPIQ7, Seattle, Washington, USA, Siemens Ultrasound Sequoia S20000, S3000, ACUSON Sequoia, Mountain View, CA, USA). A low mechanical index (<0.2) was used in all patients to prevent the unintentional destruction of microbubbles. All CEUS examinations included the intravenous application of 1.0–2.4 mL of a second-generation contrast agent (SonoVue^®^, Bracco, Milan, Italy). The application of SonoVue^®^ was followed by 5–10 mL sterile 0.9% sodium chloride solution. No side-effects upon intravenous administration of SonoVue^®^ were observed.

All examinations included native B-mode to assess tumor size, localization, shape, and echogenicity, Color Doppler to evaluate tumor vascularization, and CEUS to evaluate the dynamic contrast enhancement patterns of the lesions compared to the renal parenchyma. To determine enhancement patterns upon intravenous application of SonoVue^®^, the lesions were evaluated during the cortical phase (8–35 s after intravenous injection), the corticomedullary phase (36–120 s after injection), and the late phase (>120 s after injection to the disappearance of microbubbles). After the injection of SonoVue^®^, cine-loops were created and archived in our institutional picture archiving and communication system (PACS). Image quality was sufficient in all CEUS examinations. Radiologic reports were created by the examiner immediately after performing CEUS without knowledge of the histopathological results. 

We searched our local database for patients with suspicious renal lesions who underwent CEUS at our University Hospital. Between 2009 and 2019, 2417 patients with solid renal masses were examined. In 114 patients, CEUS was followed by either biopsy or radical or partial nephrectomy in our institutional department of urology and histopathological analysis in our institutional department of pathology. We included these 114 patients who underwent CEUS and subsequent histopathological analysis in this study. 

Statistical analysis was performed using IBM SPSS Statistics Version 25 (Armonk, NY, USA). The mean and the standard deviation (SD) were calculated for normally distributed data. Chi-squared test was performed for qualitative comparison of sonomorphologic features in native B-mode, Color Doppler, and CEUS in benign vs. malignant lesions. Statistical tests were considered significant for *p*-values < 0.05. 

## 3. Results

We included 18 patients (16%) with histopathologically confirmed benign lesions: 11 patients (10%) with oncocytoma and seven patients (6%) with angiomyolipoma. The mean age of this group at the time of the CEUS examination was 66 years (SD: ± 13 years; range: 32–81 years) with a male predominance (12 men (11%) and six women (5%), ratio: 2:1). The mean diameter of the confirmed angiomyolipomas was 2.2 cm (SD: ± 0.8 cm; range: 1.2–3.4 cm). The mean diameter of the confirmed oncocytomas was 2.3 cm (SD: ± 1.3 cm; range: 1.2–5.5 cm). 

Ninety-six patients (84%) with histopathologically confirmed renal cell carcinoma were included in this study: 47 patients (41%) with clear cell renal cell carcinoma (CCRCC), 42 patients (37%) with papillary renal cell carcinoma (PRCC), and 7 patients (6%) with chromophobe renal cell carcinoma (ChRCC). The mean age of these patients at the time of the CEUS examination was 64 years (SD: ± 12 years; range: 25 to 85 years). We included 79 male (69%) and 17 female (15%) patients (ratio: 4.6:1). The mean lesion size was 3.3 cm (SD: ± 1.7 cm; range: 0.7–10.0 cm).

**Figure 1:**
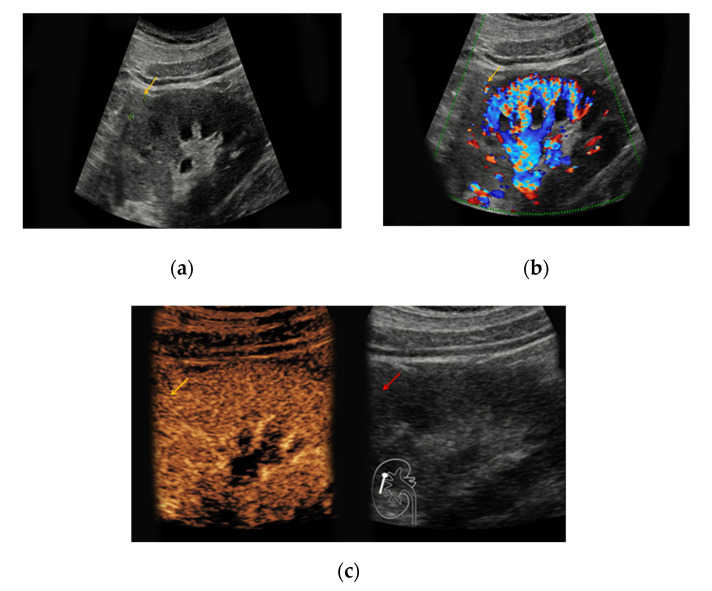
Renal oncocytoma in the right kidney with a diameter of 1.2 cm. (**a**) In native B-mode, the lesion presents as slightly hyperechoic (yellow arrow); (**b**) the lesion shows discrete vascularization in Doppler mode. (**c**) Upon intravenous application of SonoVue^®^, the lesion shows no signs of hyperenhancement.

Supplementary Table S1 illustrates the varying sonomorphological characteristics of renal oncocytomas and angiomyolipomas. No significant statistical differences between the sonomorphological features of benign vs. malignant lesions could be detected (Table 1). In native B-mode, oncocytomas presented as hypoechoic in seven cases (64%), as hyperechoic in one case (9%), and as isoechoic in one case (9%). Two lesions could not be detected in native B-mode and Doppler mode but demarcated upon intravenous application of SonoVue^®^ showing rapid contrast-enhancement (18%). Only three lesions showed slight hypervascularization in Doppler mode (27%). In all patients with histopathologically confirmed oncocytoma, early contrast-enhancement upon application of SonoVue^®^ could be detected (100%) (Figure 1). Seven of those lesions also showed venous wash-out (64%). 

**Figure 2:**
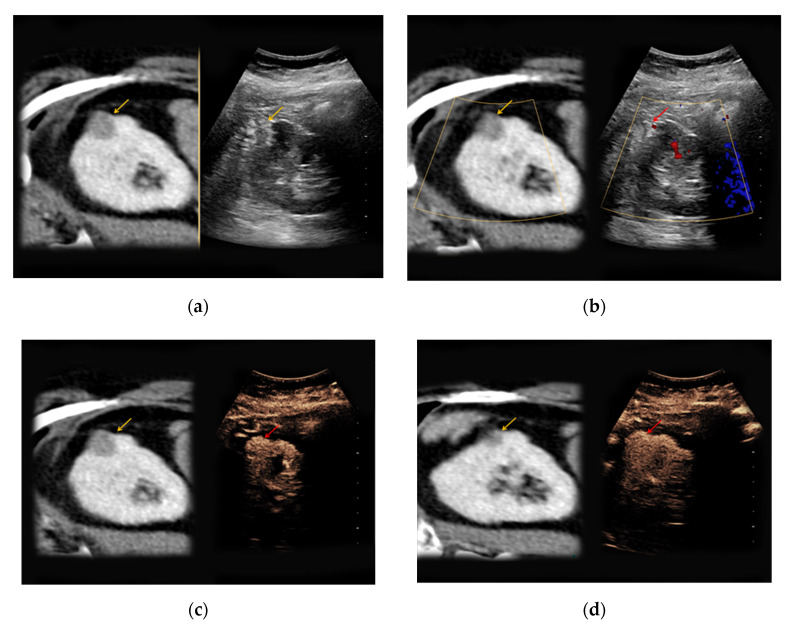
An 81-year-old patient with an unclear lesion in the right kidney incidentally detected in contrast-enhanced computed tomography (CT). (**a**) In native B-mode, an inhomogeneously hyperechoic lesion with a diameter of 2.5 cm could be registered (right picture, yellow arrow). In the corresponding contrast-enhanced CT scan (a, left picture, yellow arrow), the lesion presents hypodense with only low contrast-enhancement; (**b**) no major vascularization could be visualized in Doppler mode (right picture, red arrow). (**c**) CEUS shows a rapid hyperenhancement of the lesion (right picture, red arrow); (**d**) no wash-out in the late phase was registered (right picture, red arrow). The patient underwent partial nephrectomy, the results of the histopathological analysis revealed benign renal angiomyolipoma.

In 4/7 patients with confirmed angiomyolipoma, the lesions presented as hypoechoic in native B-mode (57%) and in 3/7 patients as hyperechoic (43%). Only one lesion showed slight hypervascularization in Doppler mode (14%). All lesions showed rapid contrast-enhancement (100%) (Figure 2), whereas only two lesions also showed venous wash-out (29%). 

**Figure 3:**
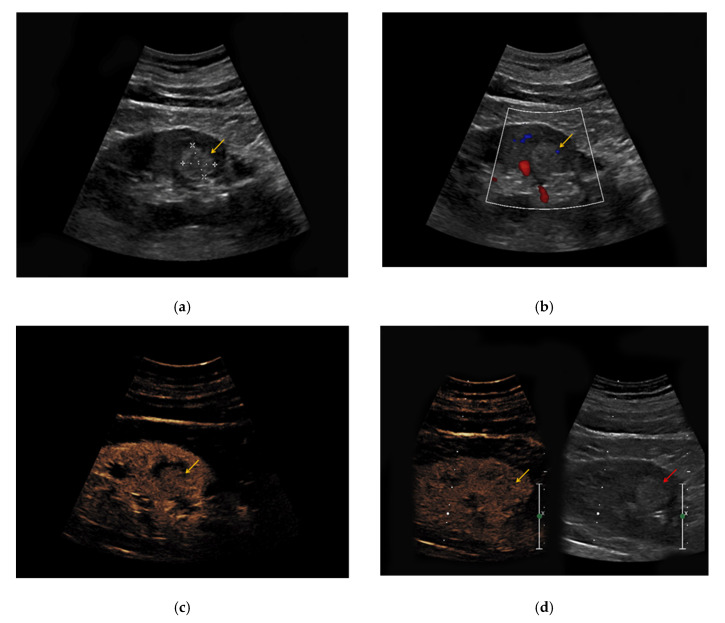
(**a**) Hyperechoic renal lesion with a diameter of 1.6 cm visualized in native B-mode (yellow arrow); (**b**) only slight vascularization was registered in Doppler mode (yellow arrow). (**c**,**d**) The lesion did not show hyperenhancement during the early (c, yellow arrow) and late phase (d, yellow arrow). Histopathological results showed a clear-cell renal cell carcinoma.

The heterogeneous sonomorphological features of the different histological subtypes of RCC are depicted in Supplementary Table S2. The majority of the CCRCC lesions presented as hypoechoic (33/47, 70%) compared to six hyperechoic lesions (13%) and five isoechoic lesions (11%). One lesion was iso-/hypoechoic (2%) and two lesions presented as hyper-/hypoechoic (4%). The PRCC lesions were hypoechoic in 27/42 cases (64%), hyperechoic in 8/42 cases (19%), and isoechoic in 5/42 cases (12%). One PRCC lesion could only be detected upon intravenous contrast application (2%) and one patient with bilateral PRCC presented with a hyperechoic lesion on the left and an isoechoic lesion on the right side (2%). Notably, 3/7 ChRCC lesions were hypoechoic (43%) compared to 4/7 isoechoic lesions (57%). Hypervascularization in Doppler Mode was detected in 7/47 CCRCC lesions (15%), in 2/42 PRCC lesions (5%), and 3/7 ChRCC lesions (43%). All 96 RCC lesions showed early enhancement upon application of contrast medium (Figure 3). Venous wash-out could be detected in 18/47 CCRCC lesions (38%), in 27/42 PRCC lesions (64%), and 6/7 ChRCC lesions (86%). 

## 4. Discussion

Non-invasive diagnosis of benign solid renal masses remains challenging since lesions such as oncocytoma and lipid-poor angiomyolipoma often show imaging features similar to RCC. Our study aimed to compare the imaging characteristics of benign and malignant solid renal masses in CEUS.

Our findings are consistent with the current state of knowledge. Several previous studies reported difficulties in the differentiation between RCC and oncocytoma using CEUS. Haendl et al. [21] described the contrast-enhancement patterns of three oncocytomas: two lesions showed arterial hypervascularization followed by venous wash-out, whereas one lesion was hypovascular during the arterial and late phase. Tamai et al. [22] evaluated 29 patients with solid renal lesions using CEUS: two patients had an oncocytoma showing arterial hypervascularization which made them difficult to differentiate from RCC. Gerst et al. [23] prospectively evaluated the imaging characteristics of benign and malignant renal lesions by CEUS, and all three oncocytomas included in this study presented as hyperechoic. Two lesions showed rapid contrast-enhancement and venous wash-out, whereas one oncocytoma showed a continuing hypoenhancement. The oncocytomas evaluated in the present study also showed various echogenicity (64% hypo-, 9% hyper-, 9% isoechoic) with rapid contrast enhancement in all lesions and venous wash-out in 7/11 lesions (64%). The imaging characteristics in our study are thus similar to the findings of these previous studies.

Similar difficulties in the discrimination between angiomyolipoma and RCC have been reported by previous studies. In the prospective study of Ascenti et al. [24], angiomyolipomas showed a wide range of characteristics concerning contrast enhancement, and the additional use of a contrast agent did not lead to higher diagnostic accuracy compared to conventional ultrasound. Other studies, however, identified imaging characteristics that might help discriminate angiomyolipoma from RCC. A retrospective study including 33 patients with angiomyolipoma and 93 patients with RCC identified venous wash-out as a feature highly suggestive of RCC (71%), which allows for differentiation from angiomyolipoma (3%) [25]. In our study, only 29% of angiomyolipomas showed venous wash-out compared to 64% of oncocytoma and 53% of RCC. Due to varying results of previous studies and our small sample size of patients with angiomyolipoma, further studies with larger patient cohorts will be necessary to evaluate the imaging characteristics of angiomyolipoma in CEUS and potentially detect reliable features which allow for safe discrimination from RCC. 

Since the diagnostic accuracy of contrast-enhanced imaging for differentiation between RCC and oncocytoma or angiomyolipoma is limited, the common treatment pathway of unclear solid renal masses frequently includes surgery in the form of radical or partial nephrectomy [5]. Henderson et al. [26] evaluated the perioperative outcomes of 6042 patients with both malignant and benign renal lesions who underwent nephrectomy in the UK, and major complications were recorded in 3.9% of all patients. Therefore, it seems desirable to allow for non-invasive diagnosis of oncocytoma or angiomyolipoma. The use of biomarkers, such as microRNAs, offers a promising approach for non-invasive characterization of unclear renal masses [27,28]. Further studies with a focus on the diagnostic accuracy of these biomarkers are needed in the future. 

The use of CEUS for assessing unclear renal lesions brings substantial advantages. CEUS is a safe imaging modality for patients with decreased renal function, hyperthyroidism, allergic predispositions to gadolinium- or iodine-based contrast agents, or contradictions for MRI, such as metallic foreign bodies [5,9,10]. Since CEUS is a non-ionizing imaging technique, its use allows for reducing exposure to ionizing radiation compared to CT. Furthermore, recent studies have described the safe application of CEUS, even in pregnant women and children [29,30]. Additionally, CEUS offers a cost-effective diagnostic approach compared to MRI [8] and could therefore reduce costs associated with medical imaging. 

Several possible limitations of our study should be considered. First, this was a retrospective single-center study and all patients were examined by only one experienced radiologist. Second, a small number of patients with oncocytoma (*n* = 11) and angiomyolipoma (*n* = 7) were included. Finally, the sample size of the different histological subtypes of RCC varied with only a small number of patients with ChRCC (*n* = 7) compared to CCRCC and PRCC. 

In conclusion, our study could not determine a specific sonomorphological feature allowing for accurate differentiation between benign and malignant solid renal masses by CEUS. Our findings are in line with several previous studies investigating the diagnostic performance of CEUS in the evaluation of malignant renal lesions and benign lesions such as oncocytoma or angiomyolipoma. Since the diagnostic workup of benign solid renal lesions often includes surgery associated with potential complications, further studies with a focus on imaging characteristics in larger patient cohorts, as well as other potential non-invasive diagnostic procedures such as biomarkers, are required.

## Figures and Tables

**Table 1:** Comparison of sonomorphologic characteristics of benign (*n* = 18) and malignant (*n* = 96) renal lesions in absolute numbers and percentages in parentheses.

	Benign	Malignant	*p*-Value
**B-mode**			
**Echogenicity**			
Hyperechoic	4 (22)	14 (15)	0.41
Hypoechoic	11 (61)	63 (66)	0.71
Isoechoic	1 (6)	14 (15)	0.30
Inhomogeneous	2 (11)	5 (5)	0.34
**Color Doppler**			
**Hypervascularization**			
Yes	4 (22)	12 (13)	0.28
No	14 (78)	84 (88)	
**CEUS**			
**Arterial hyperenhancement**			
Yes	18 (100)	96 (100)	1
No	0 (0)	0 (0)	
**Venous wash-out**			
Yes	9 (50)	51 (53)	0.81
No	9 (50)	45 (47)	

CEUS: contrast-enhanced ultrasound.

## Supplementary Materials

The following are available online at https://www.mdpi.com/1010-660X/56/11/624/s1, Supplementary Table S1: Sonomorphological characteristics of benign renal lesions, Supplementary Table S2: Sonomorphological characteristics of malignant renal lesions.
